# Revised monthly energy generation estimates for 1,500 hydroelectric power plants in the United States

**DOI:** 10.1038/s41597-022-01748-x

**Published:** 2022-11-04

**Authors:** Sean W. D. Turner, Nathalie Voisin, Kristian Nelson

**Affiliations:** 1 Pacific Northwest National Laboratory, Richland, WA USA; 2 University of Washington, Seattle, WA USA

**Keywords:** Energy supply and demand, Hydrology

## Abstract

The U.S. Energy Information Administration (EIA) conducts a regular survey (form EIA-923) to collect annual and monthly net generation for more than ten thousand U.S. power plants. Approximately 90% of the ~1,500 hydroelectric plants included in this data release are surveyed at annual resolution only and thus lack actual observations of monthly generation. For each of these plants, EIA imputes monthly generation values using the combined monthly generating pattern of other hydropower plants within the corresponding census division. The imputation method neglects local hydrology and reservoir operations, rendering the monthly data unsuitable for various research applications. Here we present an alternative approach to disaggregate each unobserved plant’s reported annual generation using proxies of monthly generation—namely historical monthly reservoir releases and average river discharge rates recorded downstream of each dam. Evaluation of the new dataset demonstrates substantial and robust improvement over the current imputation method, particularly if reservoir release data are available. The new dataset—named RectifHyd—provides an alternative to EIA-923 for U.S. scale, plant-level, monthly hydropower net generation (2001–2020). RectifHyd may be used to support power system studies or analyze within-year hydropower generation behavior at various spatial scales.

## Abstract (summary)

**Table:** 

Measurement(s)	Hydroelectric Power Generation
Technology Type(s)	Disaggregation informed by hydrological data

## Background & Summary

### Need for a new U.S. plant-level monthly hydropower generation dataset

**Fig. 1:**
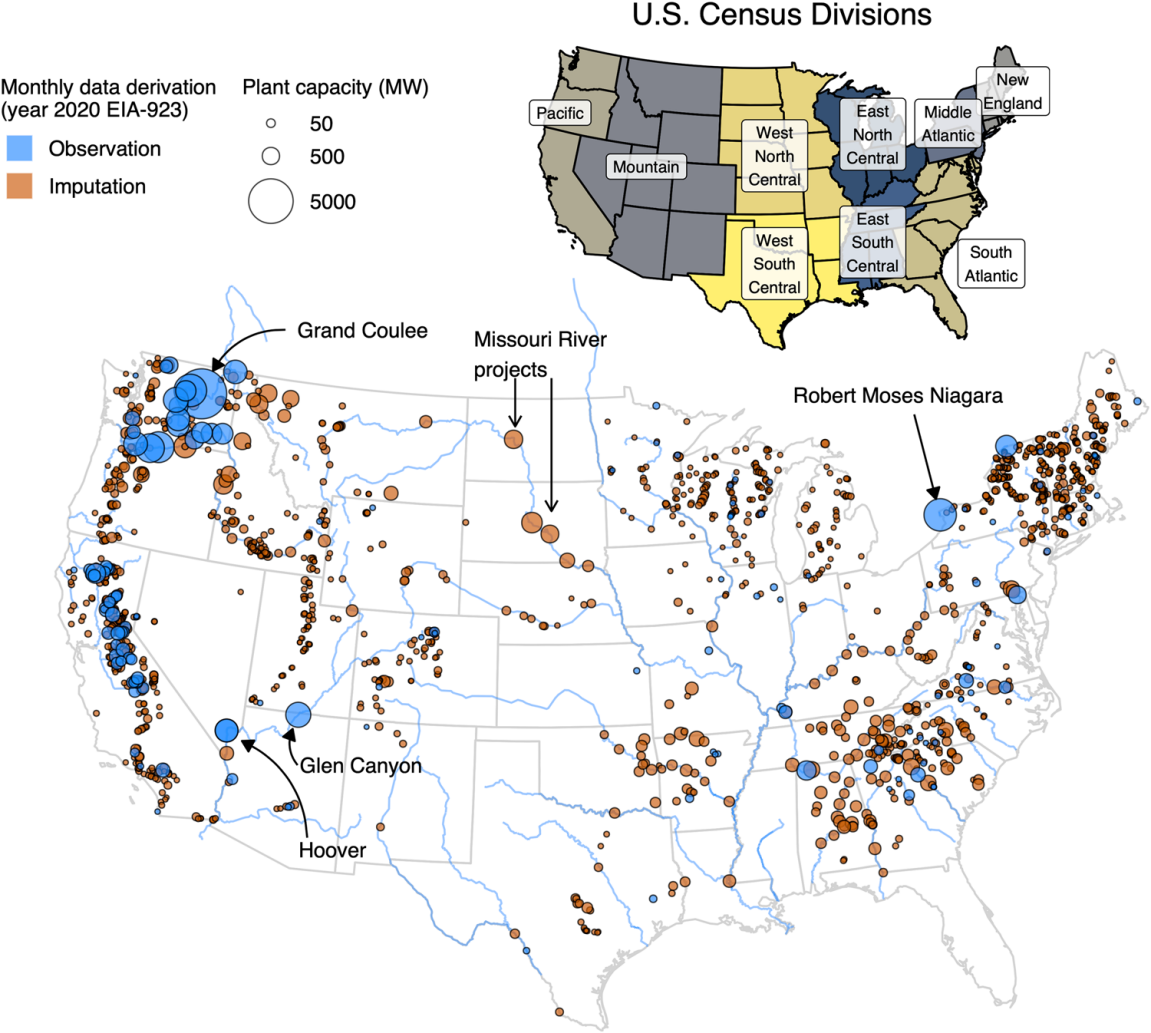
Hydroelectric power plants included in EIA-923, year 2020 submission, indicating whether monthly data are based on observations provided by utilities/operators or imputation conducted by EIA. Lower map shows U.S. Census Divisions.

Sub-annual, net generation observations for hydroelectric power plants serving the electrical grid are essential for understanding the vulnerability of electricity supply to seasonally varying water resource availability. In the United States, this data need is served by the Energy Information Administration (EIA) through its regular survey of utilities and plant operators, named form EIA-923^1^ (previously EIA-906/920). Although EIA-923 provides monthly resolution net generation for more than 1,500 hydroelectric plants, only ~150 of those plants (representing about half of total U.S. conventional hydro nameplate capacity) feature observed generation at a monthly resolution (herein *observed* plants). For the remaining plants (herein *imputed* plants), EIA collects annual net generation for each plant and then distributes that generation among months using allocation factors derived from the combined generation of observed plants within the same state^2^ (Fig. 1). In cases where a state has fewer than five observed plants, the sampling scope is expanded to multi-state regions of the U.S., known as census divisions. Importantly, this imputation is not readily apparent in supporting documentation for EIA-923, although one can decipher whether a plant is observed or imputed by the “Reporting Frequency” column in each year’s spreadsheet (“A” denotes annual, meaning the monthly data provided are imputed and not observed). Upon realizing that EIA-923 monthly hydropower net generation estimates were mostly imputed, we reverse-engineered the above imputation procedure and then confirmed the approach with EIA through email contact.

The procedure adopted by EIA is unlikely to yield realistic monthly generating patterns at imputed plants. The administrative boundaries adopted by EIA may have been selected to represent energy market regions, or regions of similarity in monthly load. But even if states and census divisions align well with those factors, the general approach would remain problematic, because these administrative boundaries do not align with regions of similarity in climate or hydrology (such as river basins). Monthly conventional hydropower generation is a function of weather and river flows above all other factors, and models adopted in industry to simulate monthly hydropower are driven by river flows rather than electricity demand^3–5^. Factors relating to the electricity market, such as load and price, affect hydropower generation at hourly or daily temporal scales but do not drive monthly total output.

**Fig. 2:**
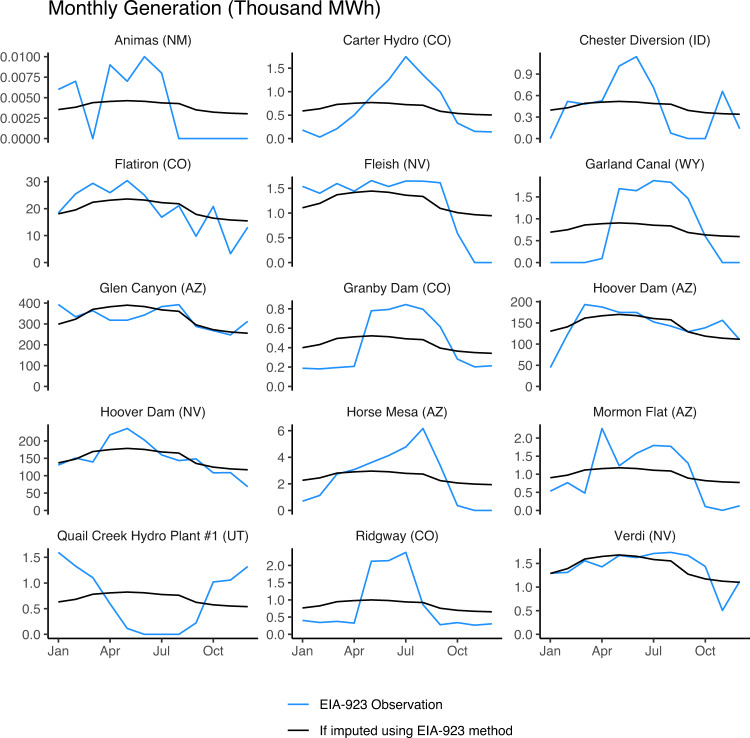
Diverse observed within-year generating behaviour in the Mountain division highlights how imputation using a single within-year shape can be inappropriate for large, multi-basin regions. The above is based on 2018 EIA-923 monthly observations.

We can demonstrate with the aid of illustrative examples that the current disaggregation approach based on states and census divisions must lead to an unrealistically smooth within-year hydropower generation pattern across many imputed plants. First, the spatial scale across which a single set of allocation factors is applied is too large. For instance, in the year 2020 EIA-923 dataset, annual generation at all 239 imputed plants across the Mountain census region (encompassing Idaho, Utah, Nevada, Arizona, Montana, Wyoming, Colorado, and New Mexico) is disaggregated to monthly resolution using just one single set of allocation factors, which are derived from a population of just 15 observed plants. Two of those plants are Glen Canyon Dam and Hoover Dam on the Colorado River—huge projects that use multi-year storage to maintain a relatively stable hydropower generation output within the year. Because the EIA’s allocation factors are based on total generation across observed plants, the largest plants dominate, leading to a relatively flat within-year shape that is unlikely to reflect generation profiles of smaller storage and run-of-river projects throughout this huge region. Run-of-river projects with low storage relative to inflow are unable to store water from one month to the next, and thus generate power with a strong seasonal signal that reflects the river flow pattern. A comparison of the monthly generation patterns for the small number of observed plants in this region reveals how unrealistic the imputed generation may be (Fig. 2).

Like the Mountain division, the observed generation of the Middle Atlantic division (New York, New Jersey, Pennsylvania) is dominated by a very flat generation profile from a large plant (Robert Moses Niagara) that is unrepresentative of generation in smaller plants in this region. Elsewhere, important cascades of projects (e.g., Missouri River, Ohio River) are unobserved at monthly scale and are imputed from a small population of small plants located hundreds of miles away. The diverse hydrological conditions within any given year across the West North Central division must result in over-smoothing as low flows (and thus below-average generation) experienced in one region cancel out high flows (above-average generation) in another, leading to monthly allocation factors that fail to represent either case.

The existing imputation procedure can be inappropriate even in regions relatively rich in monthly observational data. For instance, even though the Pacific Census region (Washington, California, Oregon) is relatively rich in monthly observations, the state of Oregon features only three observed plants. As a result, the imputed plants in this state rely on a combination of large-capacity plants on the Columbia River and smaller plants in California, located on entirely different rivers. In Washington, many of monthly observed plants are large (>1,000 MW capacity) run-of-river facilities on the Columbia River, with generation largely controlled by the spring snowmelt season and releases from Grand Coulee storage project upstream (a run-of-river project has low storage relative to river flow, perhaps one or two days flow in the case of the plants downstream of Grand Coulee; monthly generation at those plants is therefore highly correlated with river flow). These Columbia River-specific generation profiles are likely to be unsuitable to represent seasonal generation patterns of smaller tributaries to the Columbia or plants west of the Cascade Mountains toward the Pacific coast with lower reservoir storage capacities and multi-objective water uses.

The omission of local hydrology and reservoir operations from this U.S. scale hydropower dataset has important implications. EIA-923 plant-level monthly hydropower generation data are used to train grid-scale hydropower simulations^4^ and to supply seasonally varying hydropower energy budgets to power system operations models and capacity expansion models^6^. EIA-923 monthly data are also ingested without correction into other datasets, including the Existing Hydropower Assets (EHA) Net Generation Plant Database^7^ and the EIA’s state and national-scale hydropower monthly summaries available through its Electricity Data Browser. Importantly, EIA-923 hydropower data has supported a plethora of energy and water resource studies (e.g.^8–14^) as well as retrospective analyses of regional generation portfolios, which in turn inform market projections, policy analysis, and long-term planning. Users are often unaware of the limitations in the data, as it is not widely known that most monthly hydropower values in EIA-923 are not actual observations collected from utilities or plant operators. It is essential to both understand and minimize any deficiencies in the existing monthly data where possible.

In this data descriptor we introduce RectifHyd^15^—a new dataset that relies on a dramatically increased number of within-year allocation factors to estimate historical hydropower generation at imputed plants. Rather than relying on existing generation data at observed plants—which are too sparse and often unrepresentative of hydrology and operations at imputed plants—we instead develop unique allocation factors using proxy observations of plant generation, namely observed reservoir release and downstream gaged streamflow (see Methods). Reservoir release is the preferred proxy, since downstream gaged flows may be influenced by river tributaries entering between dam and gage. The latter are used when reservoir release data are unavailable. These proxies ensure that each plant is represented with a within-year generation pattern that reflects local water availability and reservoir operations. RectifHyd version 1.0 provides monthly generation estimates for the period 2001–2020.

### RectifHyd monthly generation

**Fig. 3:**
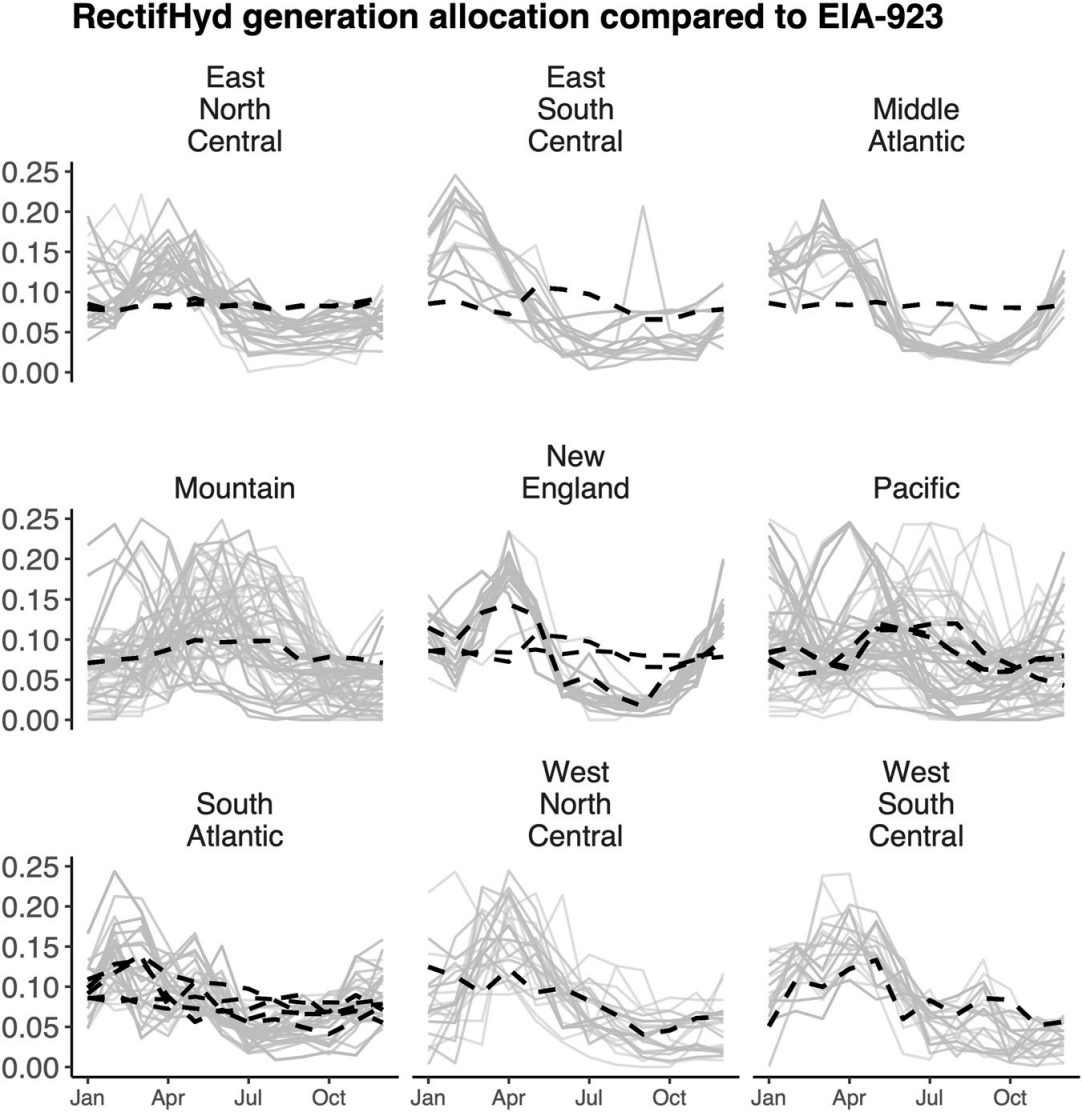
Monthly hydropower generation allocation factors applied by EIA-923 within each census division (black, broken line) compared to RectifHyd factors (grey) computed from flow proxies (year 2020 data). Census divisions with multiple EIA-923 generation factors (i.e., East North Central, New England, Pacific, South Atlantic) are those containing states with >5 observed plants, allowing state-level imputation in those cases.

RectifHyd monthly generation differs markedly from existing EIA-923 monthly imputed generation (Fig. 3). The proxy information used to infer generation in RectifHyd shows that all census divisions are characterized by far greater diversity in seasonal generation across plants than suggested by EIA-923 imputation. The most diverse hydrological conditions are found in the largest and therefore most climatically diverse census divisions—namely the Pacific and Mountain divisions. The variety of within year generating behaviors in the Mountain division shown in Fig. 2 is clear in RectifHyd generation factors, highlighting the limitations of a single, smooth monthly factors for allocating annual generation to imputed plants in this region.

**Fig. 4:**
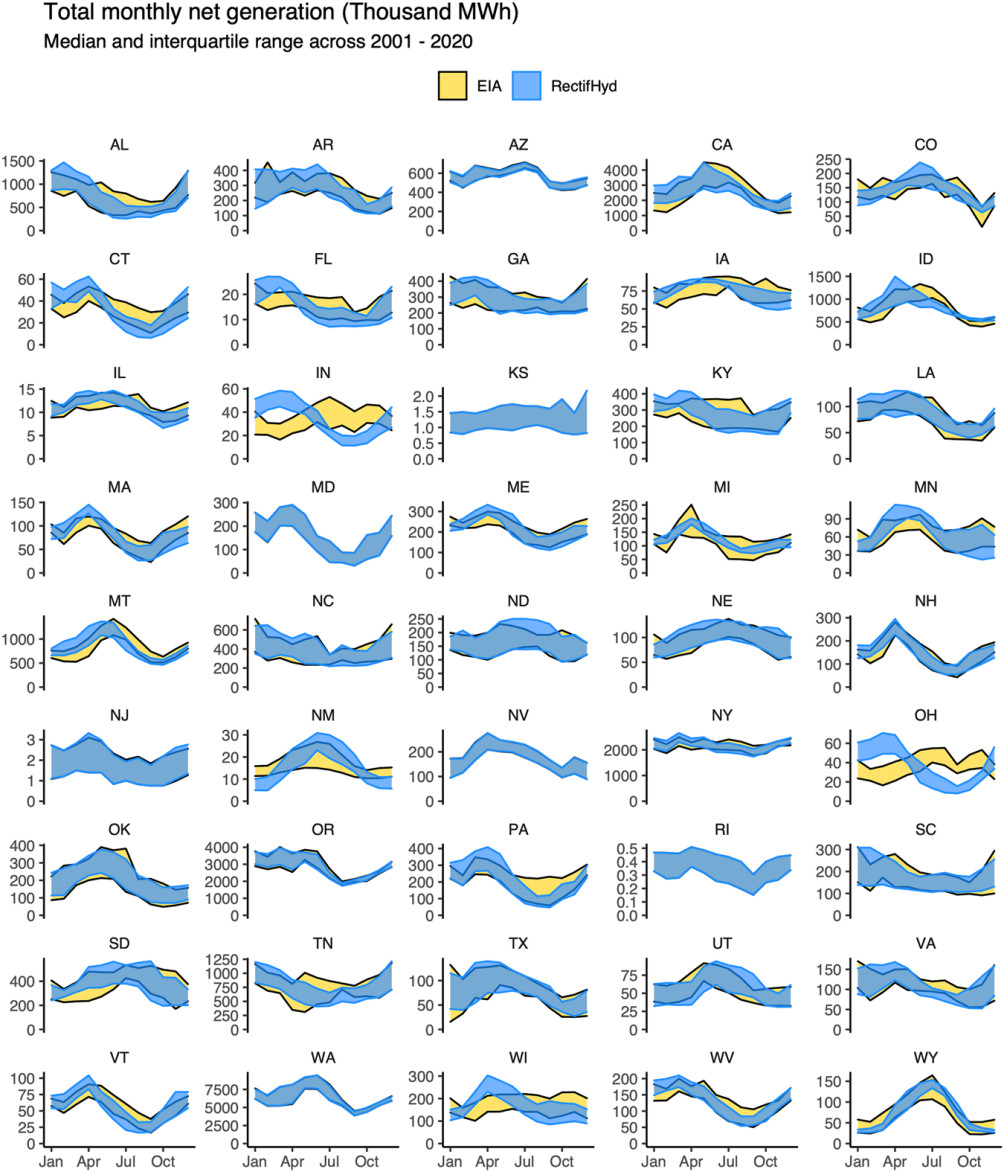
State-level monthly hydropower net generation EIA-923 compared to re-evaluated generation using RectifHyd across all years (2001–2020).

These discrepancies illustrate the relative differences between EIA-923 and RectifHyd across hundreds of plants. For some research applications, difference in the total (absolute) monthly generation will be of greater import. In a grid resilience study, for example, a 5% relative error at a 1000 MW plant may be of greater consequence than a 50% at a 5 MW plant. This may be addressed through an analysis of total absolute error across groups of plants. ReftifHyd monthly generation differs markedly from EIA-923 when compared at state-level (Fig. 4). The impact is most pronounced in states that lack hydropower generation observations, such as in the East North Central division (e.g., Indiana, Ohio) where EIA-923 generation is imputed using data observed in neighboring states. Some important hydropower generating states show significant differences in generating timing. For example, in Idaho (ID) RectifHyd shows a slightly earlier peak and overall sharper hydrograph, highlighting the deficiencies of Mountain division imputation affected by stable generation at Glen Canyon (AZ) and Hoover (NV/AZ) discussed previously. The impact is less pronounced in states where observed plants account for a relatively large share of generating capacity, such as in Washington, California, and Arizona.

**Fig. 5:**
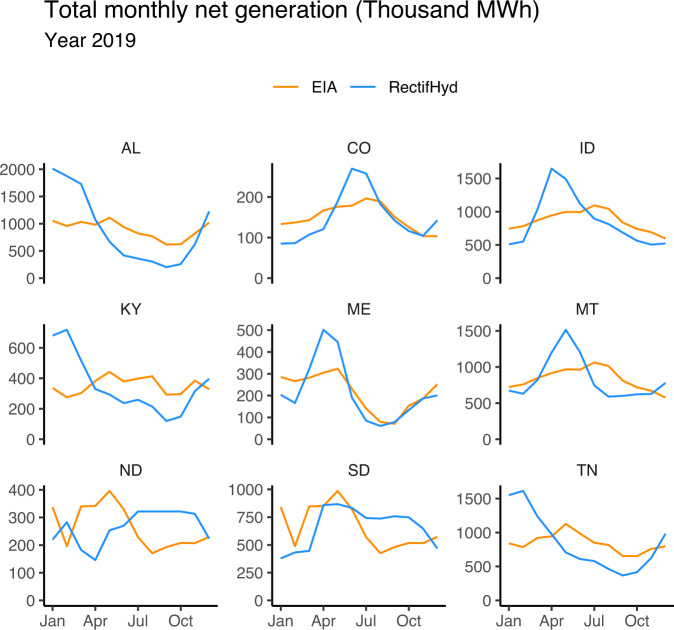
Year 2019 state-level monthly hydropower net generation EIA-923 compared to re-evaluated generation using RectifHyd across.

Discrepancies between EIA-923 and RectifHyd state-level monthly generation are often more apparent when specific years are isolated (Fig. 5). In year 2019, for example, there is large disparity in generation timing in several states where hydropower contributes significant generation, including data-sparse states like North Dakota (ND), South Dakota (SD), Kentucky (KY), and Colorado (CO). Both Alabama (AL) and Tennessee (TN) total monthly generation follow significantly smoothed pattern in EIA-923 relative to RectifHyd.

## Methods

### Overview

**Fig. 6:**
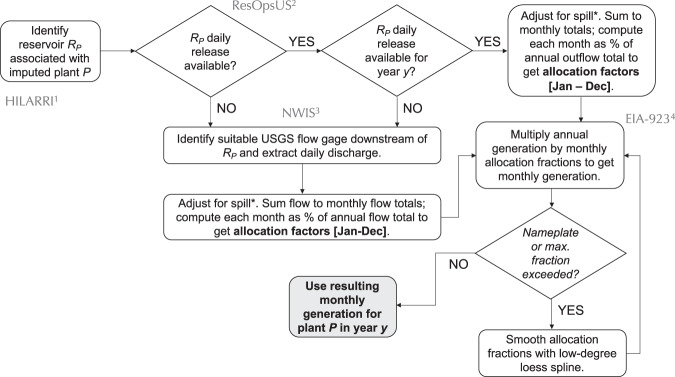
Method flow diagram for RectifHyd monthly allocation of annual hydropower generation at an imputed plant *P* in year *y*. Key input datasets are identified in grey text—namely (1) the Hydropower Infrastructure Lakes, Reservoirs and Rivers (HILARRI) dataset^17^, (2) ResOpsUS^19^, (3) U.S. Geological Survey National Water Information System^20^; and (4) EIA-923 observed annual generation totals^1^. *Adjustment for spill caps reservoir release at the 90^th^ percentile of the daily time series.

To disaggregate observed annual net generation at an imputed plant we adopt the procedure outlined in Fig. 6. In brief, we rely on one of two proxies of monthly generation at each plant: observed reservoir release time series, and, if release is unavailable, streamflow recorded downstream of the reservoir. Hydroelectric dams produce power by releasing water through turbines, so data describing those releases or describing downstream flow that is influenced by those releases should, in theory, provide an excellent proxy for how generation varies across months of each year. Reservoir release is the better of the two proxies for generation, since these flows are unaffected by tributary inflows. Yet even reservoir release is imperfect, because a portion of release may often be non-powered release (controlled or uncontrolled spill), and because generation per volume of water released through a turbine varies with reservoir head level. We shall return to these limitations in Technical Validation.

The reservoir release and downstream flow records used to create RectifHyd are extracted at a daily temporal resolution (see data sources in Sections 2.2 and 2.3, respectively). To account for turbine flow capacity and possible non-powered spill during high flows, we cap the daily flow time series at Q_90_ (90^th^ percentile of daily time series over the period 2001–2020). This threshold could, in future work, be adjusted for individual dams with the availability of either historical release time series that separate powered from non-powered flows or data describing penstock and turbine capacities across a large sample of dams. We then aggregate capped daily flows to monthly flow volumes and use these to compute a provisional set of monthly energy allocation factors for each plant. Each month’s allocation factor in each year is equal to the total monthly flow volume divided by total annual flow volume. These factors are provisional because, in some instances, they require further adjustment due to unrealistic peaking behavior that implies violation of plant capacity as reported in the Existing Hydropower Assets database^16^. One possible reason for this error is tributary flow; if the downstream gage is influenced by a tributary with unregulated flow then the proxy may deviate significantly from the seasonal power generation pattern. Another possible reason would be if spill is underrepresented by the Q_90_ assumption described above. If this error is present, we apply a loess smoothing spline to the provisional allocation factors (low degree of smoothing; span = 0.2), repeating until generation is more reasonable with nameplate capacity not exceeded and no monthly factor greater than 0.25, which would imply a quarter of annual generation occurring in a single month. This threshold was selected based on an analysis of existing monthly generating data across observed plants (0.25 is very rarely exceeded). Final adjusted factors are then multiplied by observed annual generation to create a rectified set of monthly generation estimates that sum to observed annual generation at each plant.

RectifHyd provides these monthly generation estimates for approximately 1,500 hydropower plants for the twenty-year period 2001–2020 (number of plants varies marginally each year, based on EIA-923 data availability). We opt to start RectifHyd in 2001 because this year is associated with a significant increase in the number of plants captured in the EIA survey (prior to 2001 the EIA data are provided in a different format, with approximately 300 fewer hydropower plants than the 2001–2020 data; for the years 1970–2000 the EIA collected all hydropower data at monthly resolution, meaning no statistical imputation is required for these data). RectifHyd also includes the original EIA-923 monthly generation data and a column indicating whether these are observed or imputed, allowing the user to retain EIA-923 data points for observed plants and adopt RectifHyd for others. All code used to create RectifHyd is made available openly on a Github meta-repository (see Code Availability).

### Reservoir release proxy

To determine whether a hydropower plant is associated with publicly available reservoir release record, we first connect EIA plant identifiers to reservoir identifiers using the Hydropower Infrastructure Lakes, Reservoirs and Rivers (HILARRI) dataset^17^. Specifically, we use the Global Reservoir and Dams (GRanD)^18^ identifier for each reservoir, which allows us to connect these data to reservoirs included in the ResOpUS database^19^. ResOpsUS is the most comprehensive reservoir operations dataset available for CONUS, covering historical reservoir inflows, release, and storage volumes, with more than 679 major reservoirs included. We identify 180 reservoirs with suitable release data to inform allocation factors in RectifHyd. Many reservoir release records in ResOpsUS terminate mid-2020, meaning the records are unavailable for allocating energy for the final year of available EIA-923 data. For this reason, our disaggregation procedure is conducted separately for each year, allowing the proxy being used for a plant to switch from reservoir release to downstream gaged flow if reservoir release data are missing or unsuitable for any particular year.

### Downstream flow proxy

Approximately 1,200 imputed plants lack publicly available reservoir operations records. For these plants we use downstream United States Geological Survey (USGS) gaged discharge as proxy for generation^20,21^. Some plants are associated with reservoirs that have a USGS flow gage directly downstream of the dam, but the vast majority do not. Furthermore, many gages that are in suitable locations downstream of plants lack a complete record of flow over the last two decades. In general, there are too few USGS gages to allow for all 1,200 imputed plants to be represented with a unique and ideal regulated discharge record.

To overcome these challenges, we adopt a practical, simplified approach in which we manually identify a suitable proxy gage for groups of plants rather than all individual plants. Plants are first grouped according to their USGS Hydrologic Unit Code 4 (HUC4) watersheds. There are 150 distinct HUC4 watersheds with conventional hydroelectric power plants in the United States; we identify the best proxy gage for hydroelectric generation in each of these HUC4 watersheds using visual inspection and expert judgement. Specifically, we overlay plant locations, USGS gage locations, and the NHDPlusV2 flow accumulation raster to identify (by visual inspection) a flow gage that lies downstream of the majority of hydroelectric power plants in the HUC4 and with minimal interference from unregulated streams between power plants and flow gage. We exclude observed hydroelectric power plants from this manual search, ensuring that flow gages used for imputation are most relevant to the plants being imputed. Gages with gaps of more than a few days in the discharge record are avoided.

## Data Records

RectifHyd data described in this paper have been deposited at Zenodo under accession code 6607824^15^ and can be downloaded in.csv format from https://zenodo.org/record/6607825#.YplTvi-cZTZ.

## Technical validation

### Dual evaluation approach

RectifHyd is evaluated using two distinct approaches. We first evaluate using all of the 127 observed plants in EIA-923. As with the imputed plants, we compute monthly generation for each observed plant using the best available proxy, and then compare against observed generation. We use the Kling Gupta Efficiency (KGE), Nash Sutcliffe Coefficient (NSE), and R-squared to evaluate generation at each plant. Prior to 2013, EIA did not include information on which plants were imputed for monthly generation. This evaluation is therefore based on 2013–2020 monthly generation only.

Of the 127 observed plants in EIA-923, 31 are associated with a reservoir release record in ResOpsUS. The remaining 96 observed plants must rely on the downstream flow based proxy. As noted above, USGS gages adopted in RectifHyd are selected to best represent imputed plants. This means that validation scores (KGE, NSE, R-squared) based on observed plants likely underestimate the performance of the imputation using flow. This first evaluation is primarily used to confirm expected behavior in RectifHyd data and to compare performances across the two alternative proxies. The evaluation is limited by the relatively small number of observed plants and their concentration in a small number of states across the U.S. (primarily in Washington and California).

Importantly, there are major limits to what can be learned by comparing this evaluation to a similar evaluation of the EIA-923 imputation method over the same 127 plants. The EIA-923 method relies on the same generation observations against which the model would be evaluated. Performance metrics computed for those sites would not reflect performance metrics for imputation. A “leave one out” validation of EIA-923 imputation is possible but would be misleading and uninformative. This is because observed plants are often grouped within regions that are similar hydrologically. For example, the EIA-923 factors derived for the state of Washington may perform well when analyzed against other observed plants, since nearly all observed plants are located on the Columbia River. A leave-one-out analysis would provide no indication of the performance of those factors when used to disaggregate plants across other parts of Washington.

To compare the EIA-923 imputation performance against the proxy-based approach adopted in RectifHyd, a second evaluation is performed using EIA data for the period 1970–2000. These data include approximately 300 fewer plants than EIA-923 post 2000, but all monthly observations are observed (not imputed), providing a relatively large sample of plants with which to perform an evaluation. This evaluation cannot be conducted on all plants included in the 1970–2000 monthly generation dataset, since proxy information selected for RectifHyd does not extend back into the 20^th^ century in all cases.

To replicate the existing 923 imputation method for this evaluation, we first isolate plants that are observed in the latter EIA-923 dataset (2001–2020). Generation from these plants is summed to census division monthly totals and divided by annual generation to determine the allocation factors. Those allocation factors are then multiplied by observed annual generation at each imputed plant (n = 946), following the same procedure used in EIA-923. Once plants lacking suitable proxy information are removed, this leaves 946 plants, of which 132 can be proxied using reservoir release and 814 using the downstream flow information gathered to create RectifHyd.

### Results for evaluation using 127 observed plants

**Fig. 7:**
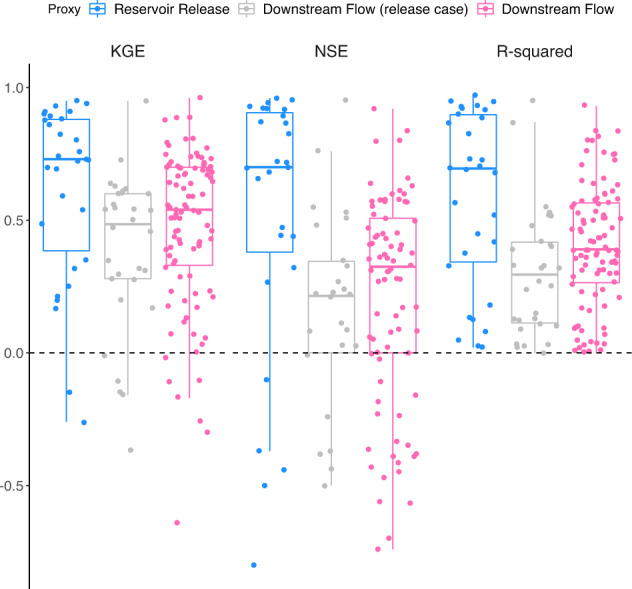
Evaluation of RectifHyd on observed plants using reservoir release proxy (n = 31), downstream flow proxy applied to release cases (n = 31), and downstream flow proxy (n = 96). Results for the downstream flow proxy may underestimate RectifHyd performance, since flow gages are selected to best represent imputed rather than observed plants.

Evaluation of RectifHyd against observed monthly generation shows that monthly reservoir release is a significantly better proxy of plant generation than monthly downstream flow (Fig. 7). Results show median KGE of 0.74, 90% confidence interval [−0.07, 0.93] across plants with observed reservoir release, and KGE of 0.51 [−0.28, 0.79] for cases imputed using a downstream flow gage. Similar differences are found for NSE and R-squared metrics. The impact of proxy type is also apparent when the plants that benefit from a release data have the proxy switched to downstream flow, which results in a significant degradation in performance.

Some observed plants in this evaluation are allocated inaccurate generation due to the selected proxy flow gage being inappropriately sited (recall the aim of RectifHyd is to disaggregate imputed plants, not observed). For instance, the three observed plants in Oregon (Bonneville, John Day, and The Dalles) are all located on the Columbia River at the downstream end of HUC 1707 (“Middle Columbia”). The imputed plants in this HUC4 are located on tributaries to the Columbia, including on the Deschutes River. Thus, the selected flow gage for imputation (14103000 Deschutes River at Moody, Near Biggs, OR) is appropriate for disaggregating generation at those plants but not for the three observed plants in this HUC4. For the evaluation at power plant with observed monthly generation, we did not adjust the siting of the representative USGS gage—resulting in a likely underestimate of performance of for the downstream flow proxy method. Plant-level time series for all observed plants are included in Supplementary Figures.

### Results for evaluation using 1970–2000 monthly observations

**Fig. 8:**
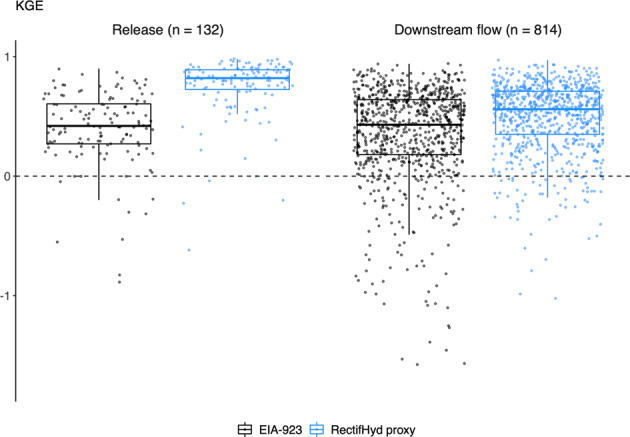
KGE scores for monthly hydropower created for period 1970–2000, comparing EIA-923 imputation method against RectifHyd proxy for the reservoir release cases (132 plants) and downstream flow cases (814 plants).

RectifHyd outperforms the EIA-923 method when evaluated using 1970–2000 monthly observations, with the reservoir release proxy providing the largest and most robust improvement (Fig. 8). The reservoir release proxy achieves median KGE of 0.82, 90% confidence interval [0.26, 0.96], based on results for 132 plants. This compares to KGE of 0.42 [−0.19, 0.79] for the EIA-923 method applied to the same 132 plants. The downstream flow proxy achieves median KGE of 0.56 [−0.12, 0.84], based on results for 814 dams. This compares to KGE of 0.43 [−0.44, 0.80] for the EIA-923 method applied to the same plants.

**Fig. 9:**
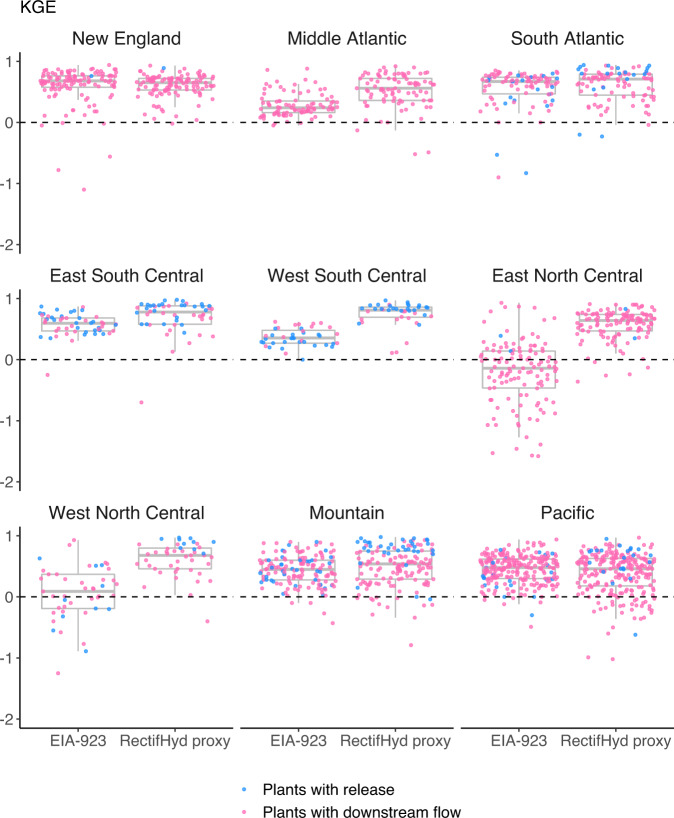
KGE scores for monthly hydropower created for period 1970–2000, for which observational data are available, comparing EIA-923 imputation method against RectifHyd for each census region.

The RectifHyd approach achieves robust performance improvement over EIA-923 for most census regions (Fig. 9). Largest and most robust performance improvements are found in regions with relatively few plants represented as well as regions where most plants can be associated with a reservoir release record to inform disaggregation of annual generation. RectifHyd does not outperform EIA-923 imputation method in the Pacific or New England census regions overall for the plants evaluated. These are regions featuring hundreds of hydropower facilities and, especially for New England, limited reservoir release data for a generation proxy. RectifHyd could perhaps be improved in these regions if a larger number of downstream flow gage information; one gage selected per HUC4 watershed may be insufficient in these regions to represent the large diversity of hydropower plants located across many and diverse rivers. Strong performance of reservoir release proxy in all regions shows how further collection of reservoir operations data could be employed to drastically improve the accuracy of available monthly hydropower generation data for the United States.

## Supplementary information

Supplementary Information

## Data Availability

As part of the Integrated Multisector, Multiscale Modeling (IM3) project (funded by the U.S. Department of Energy’s Office of Science; see “Acknowledgements”), we are committed to delivering all research products as open-source to benefit those who may have interest in reproducing or building off of our work. For the reader’s convenience, we provide the open-source code as well as step-by-step documentation for how to reproduce RectifHyd in the following meta-repository: https://github.com/immm-sfa/turner_voisin_nelson_2022_scientific_data.
