# Supplementary material for: Revised monthly energy generation estimates for 1,500 hydroelectric power plants in the United States
Source: Sci Data. 2022 Nov 4;9:675. doi: 10.1038/s41597-022-01748-x (PMC9636176; doi:10.1038/s41597-022-01748-x)

Supplementary Information for “Monthly generation estimates for 1,500 hydroelectric power plants in the United States”

Sean Turner, Nathalie Voisin, Kristian Nelson

Contents

Validation for disaggregation based on reservoir release …page 5.

Validation for disaggregation based on downstream flow …page 8.

Model validation (release-based)

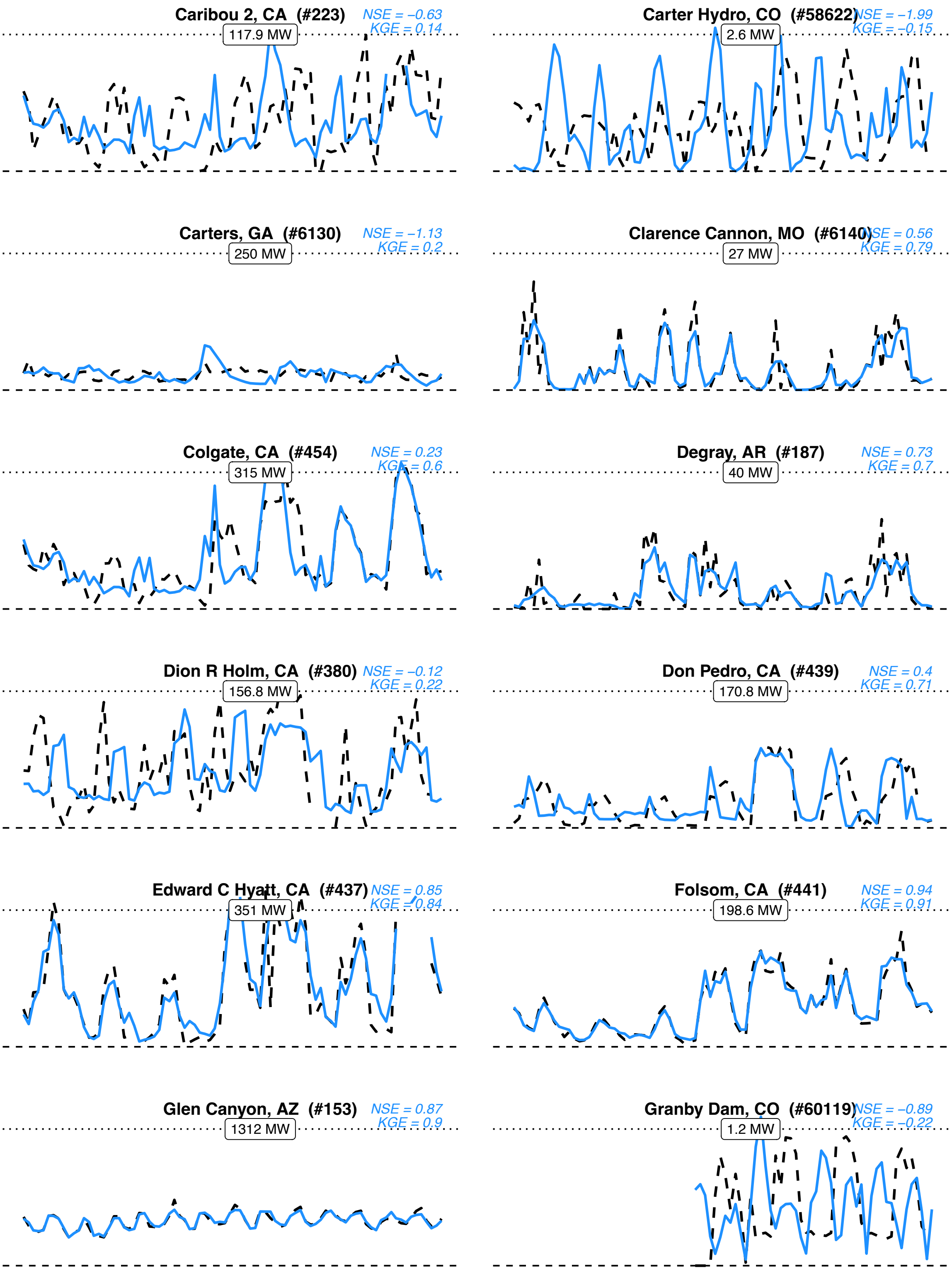

Model validation (release-based) continued.

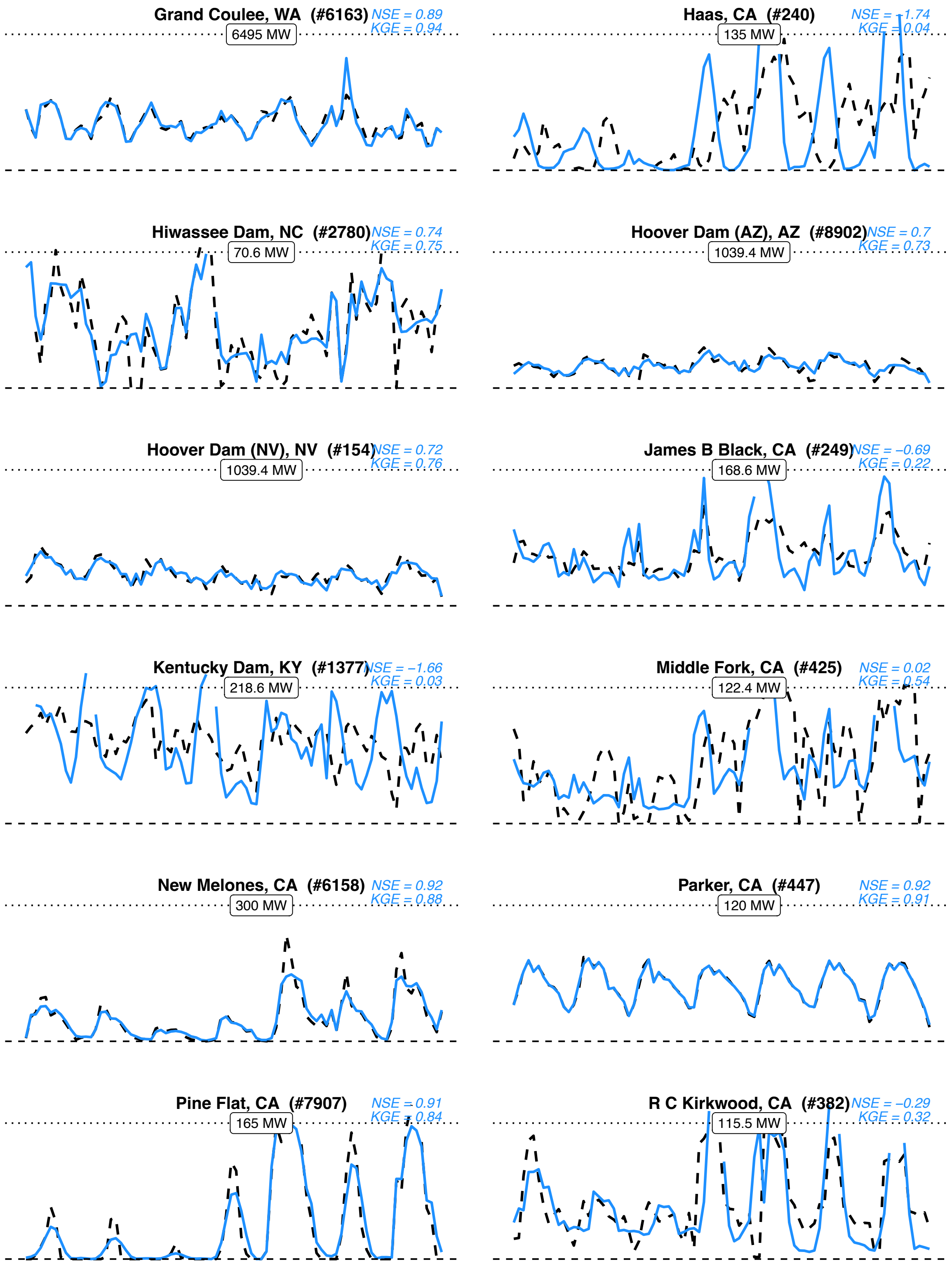

Model validation (release-based) continued.

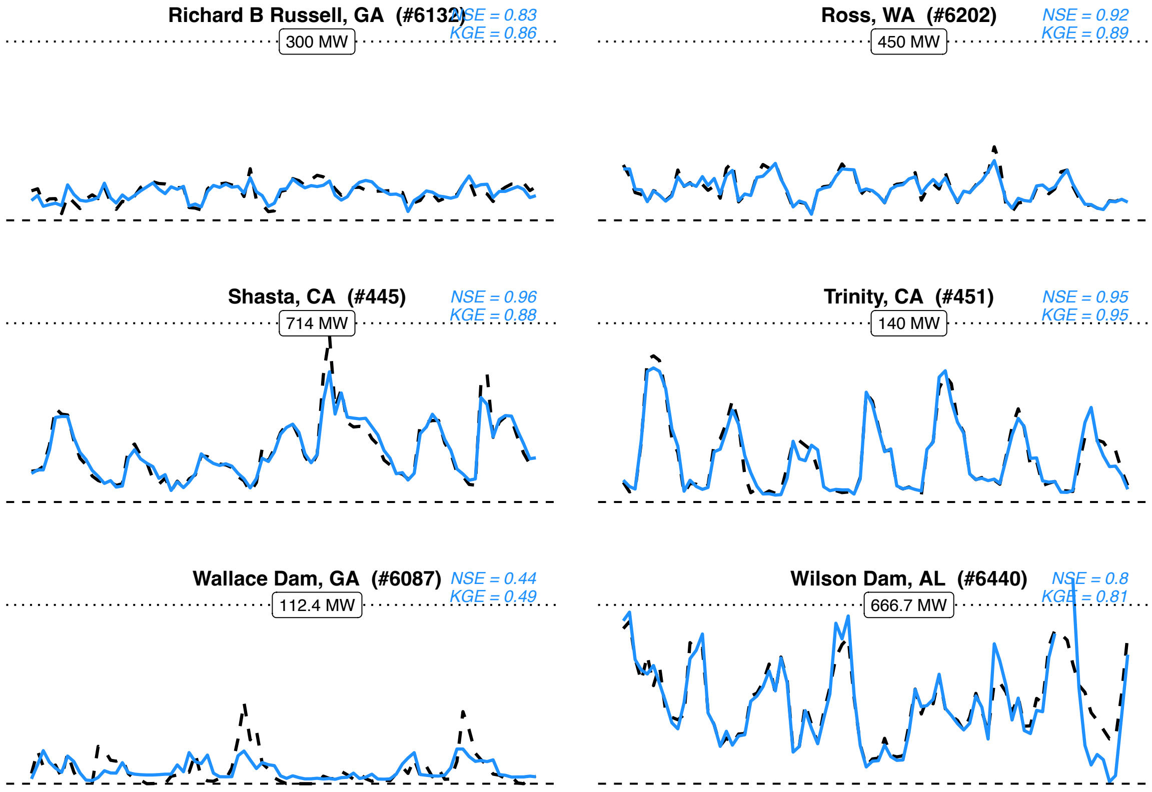

Model validation (downstream flow based)

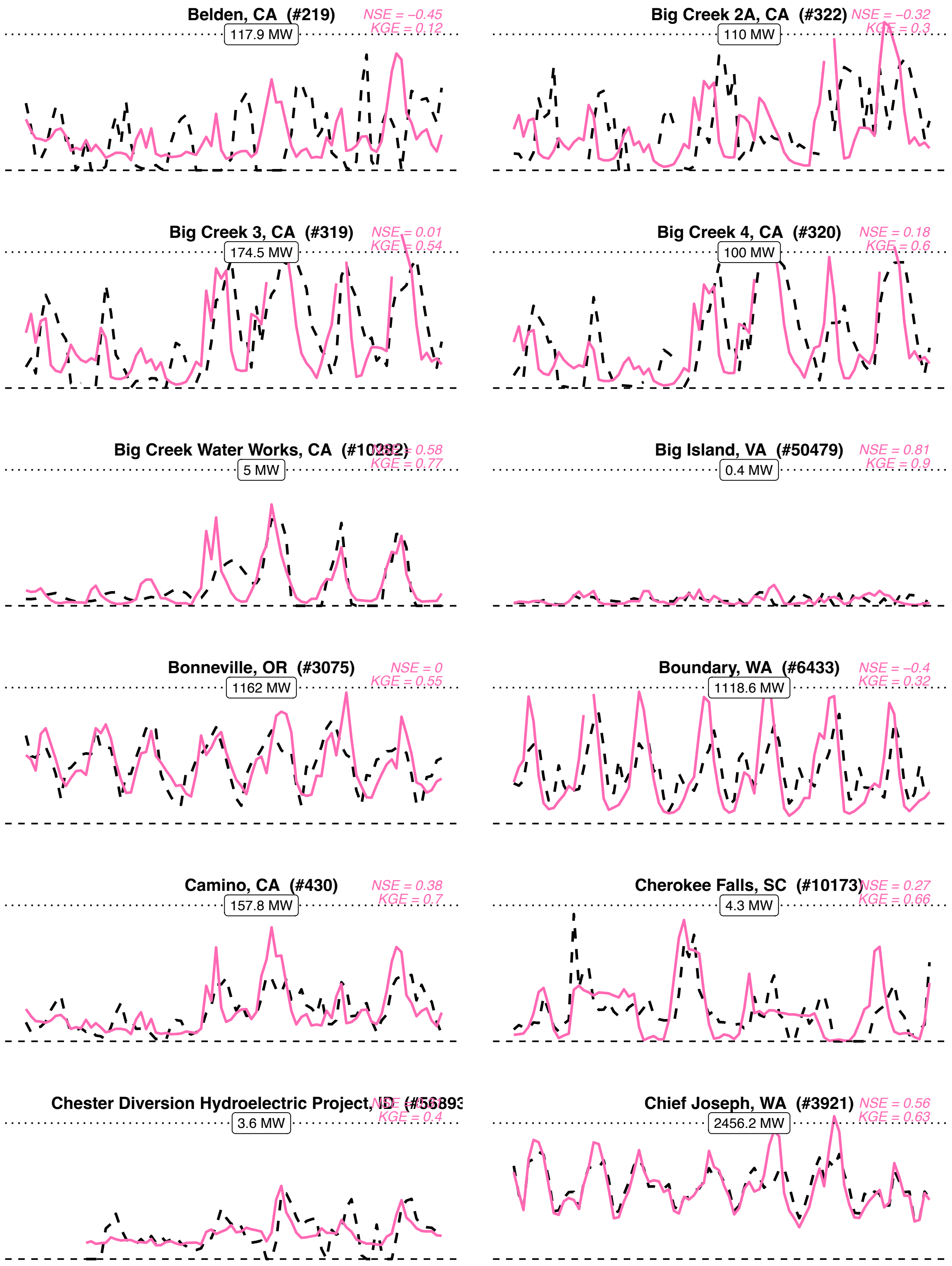

Model validation (downstream flow based) continued.

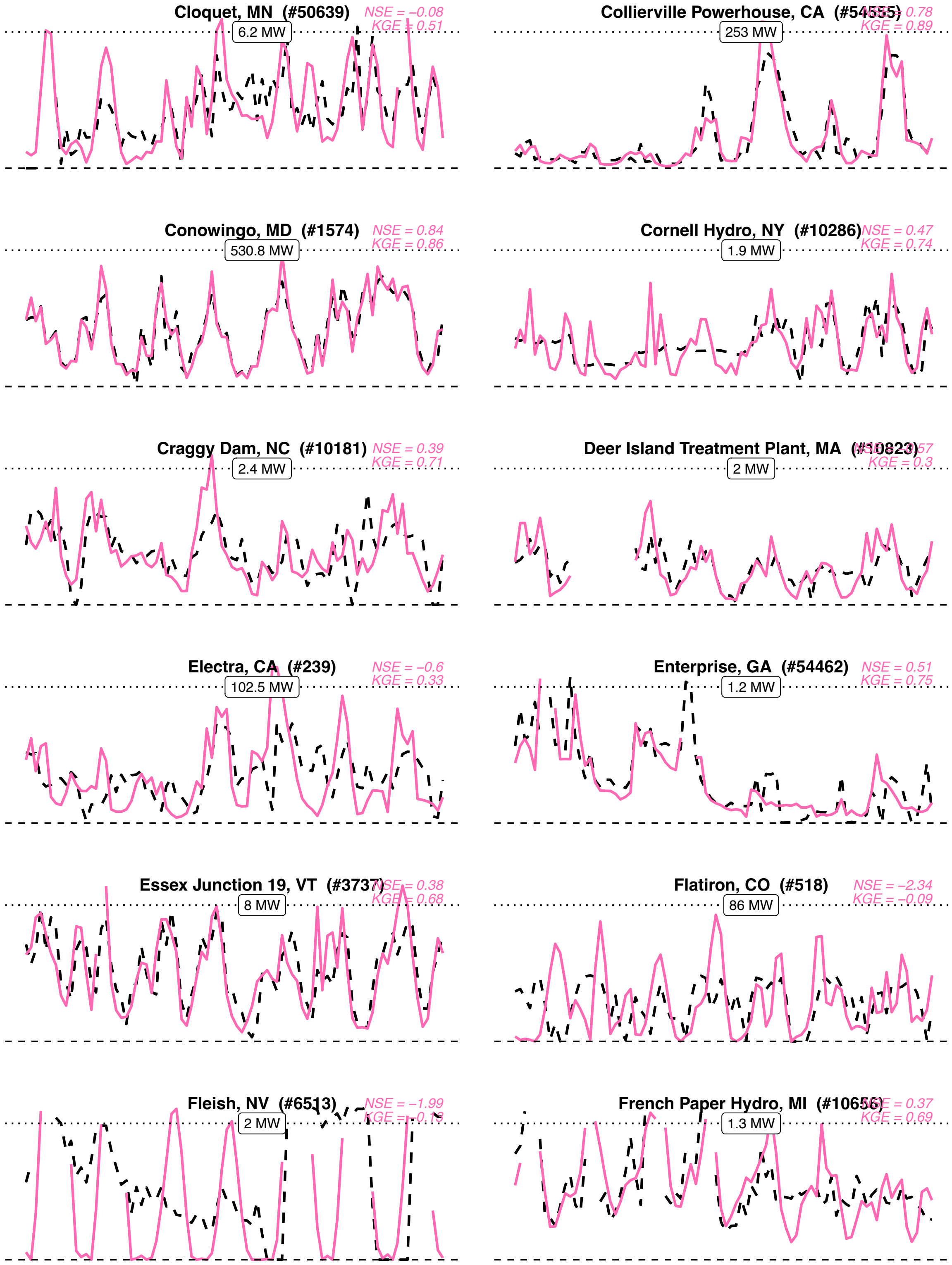

Model validation (downstream flow based) continued.

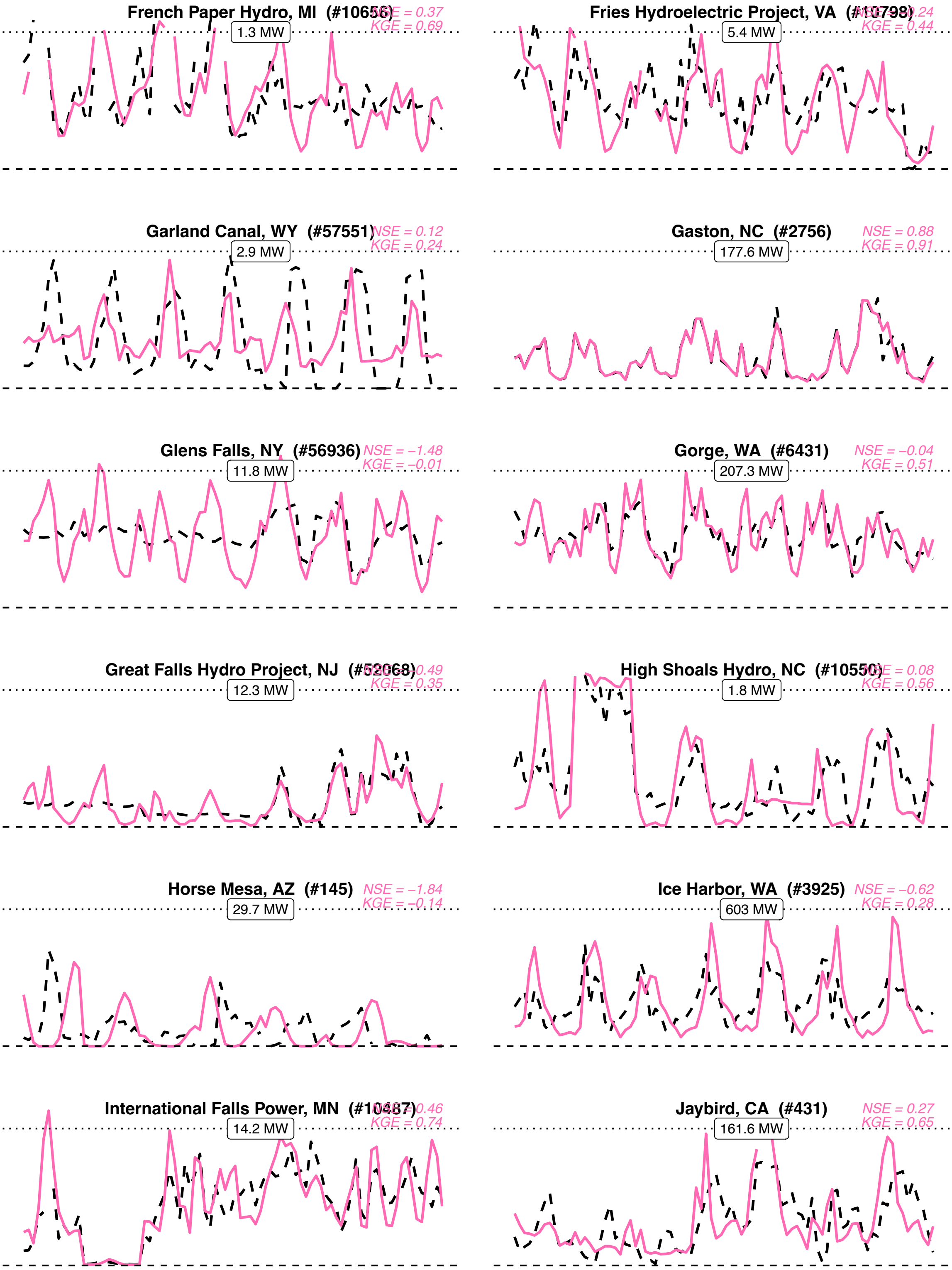

Model validation (downstream flow based) continued.

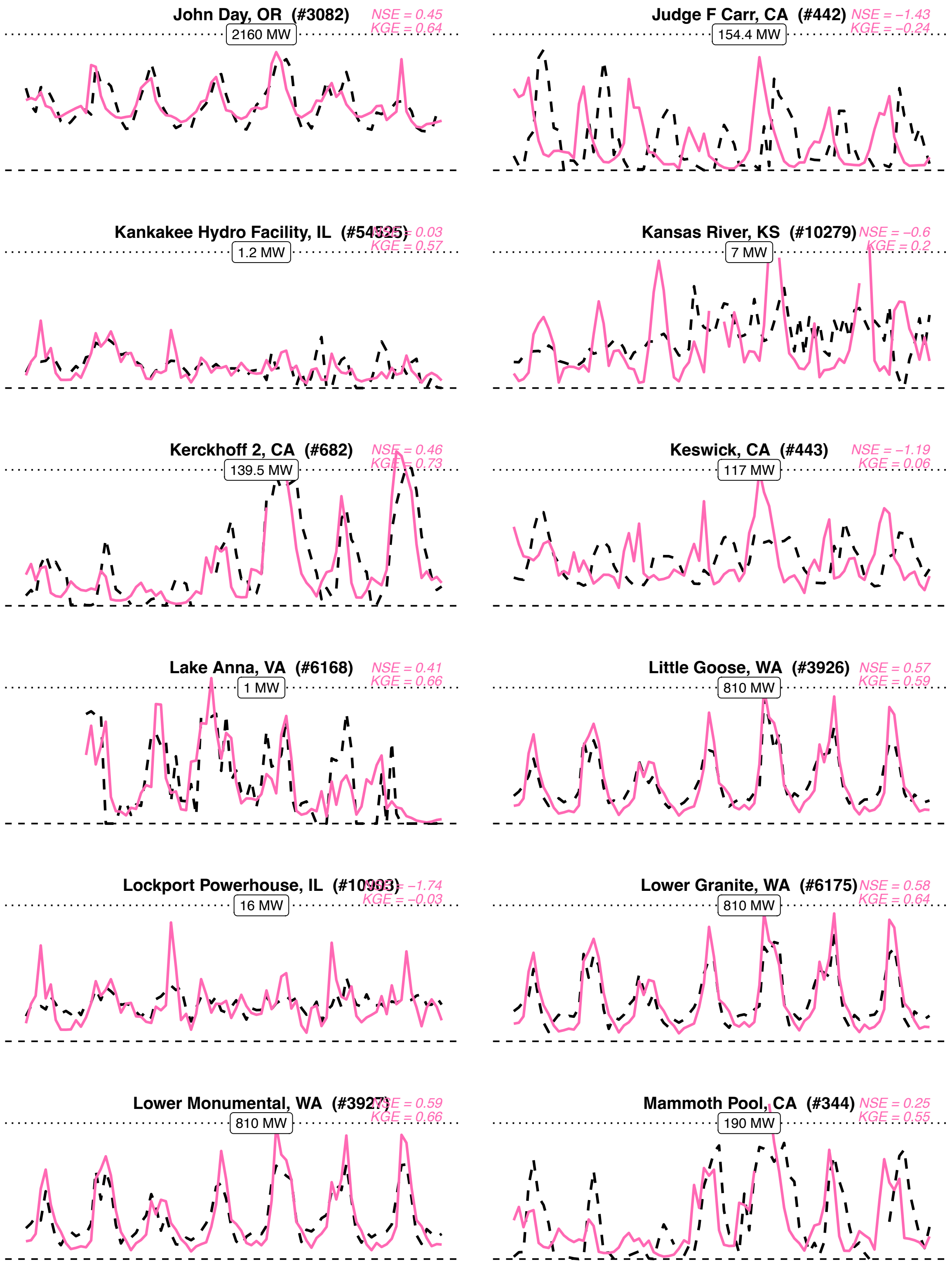

Model validation (downstream flow based) continued.

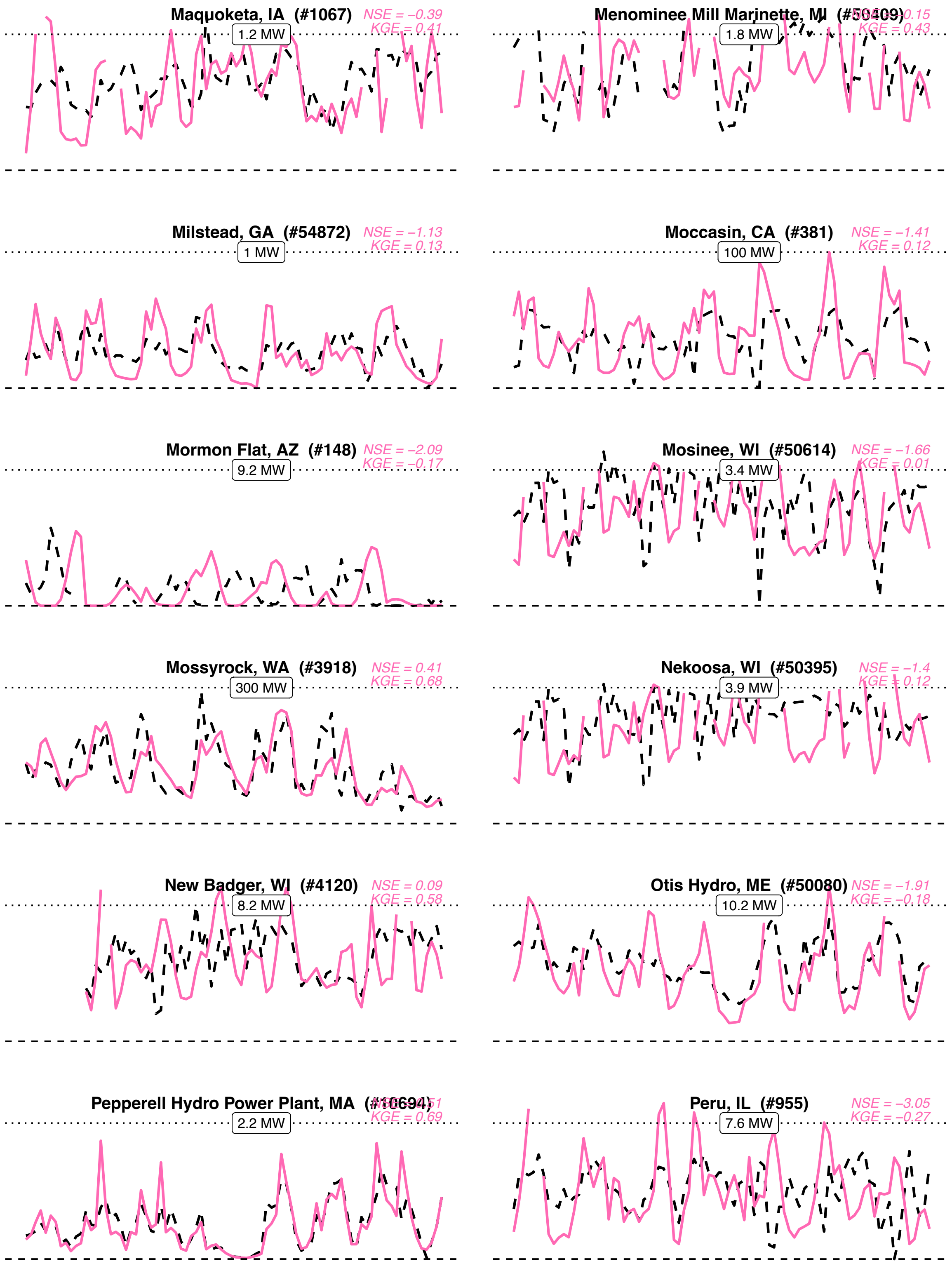

Model validation (downstream flow based) continued.

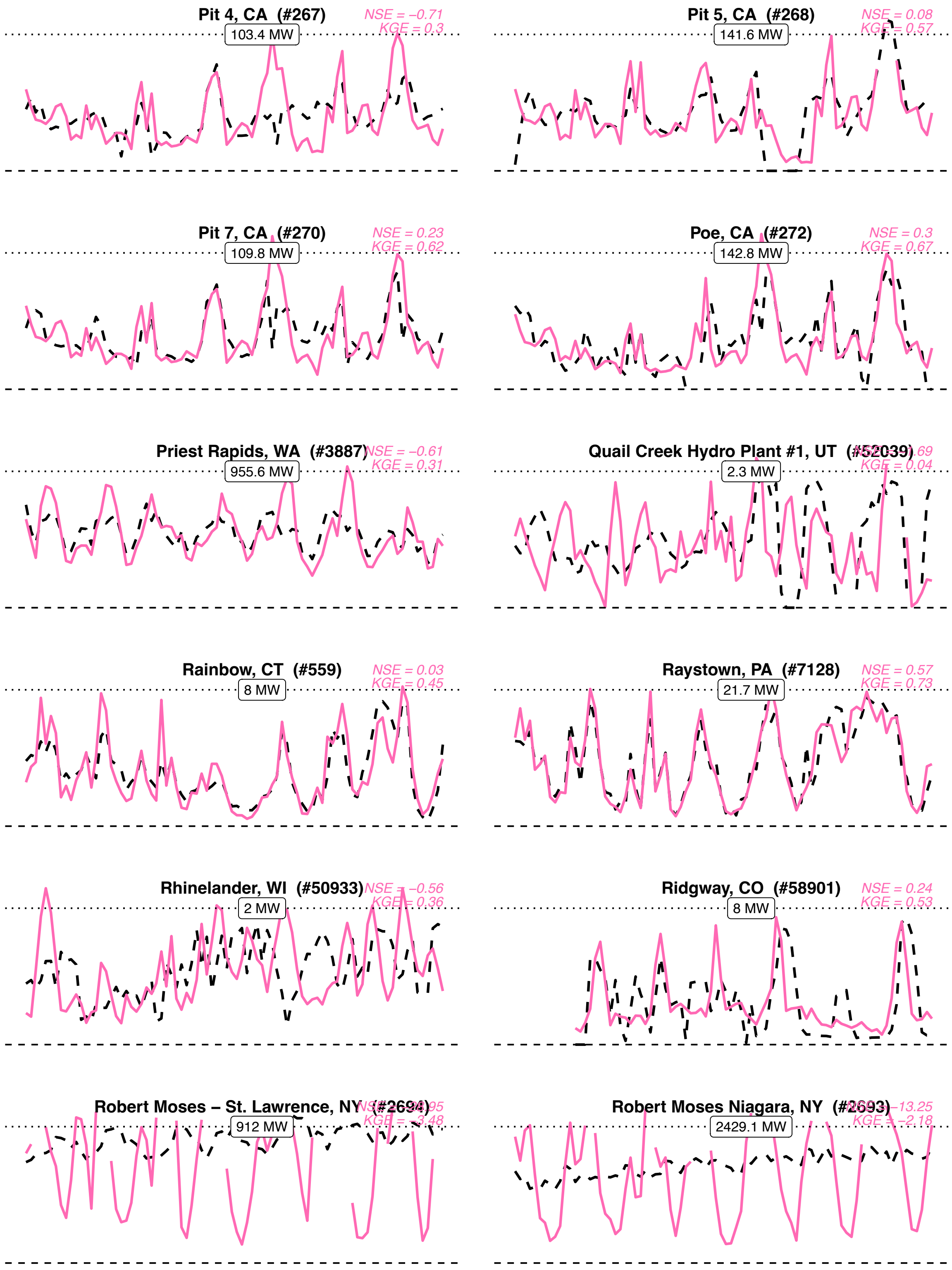

Model validation (downstream flow based) continued.

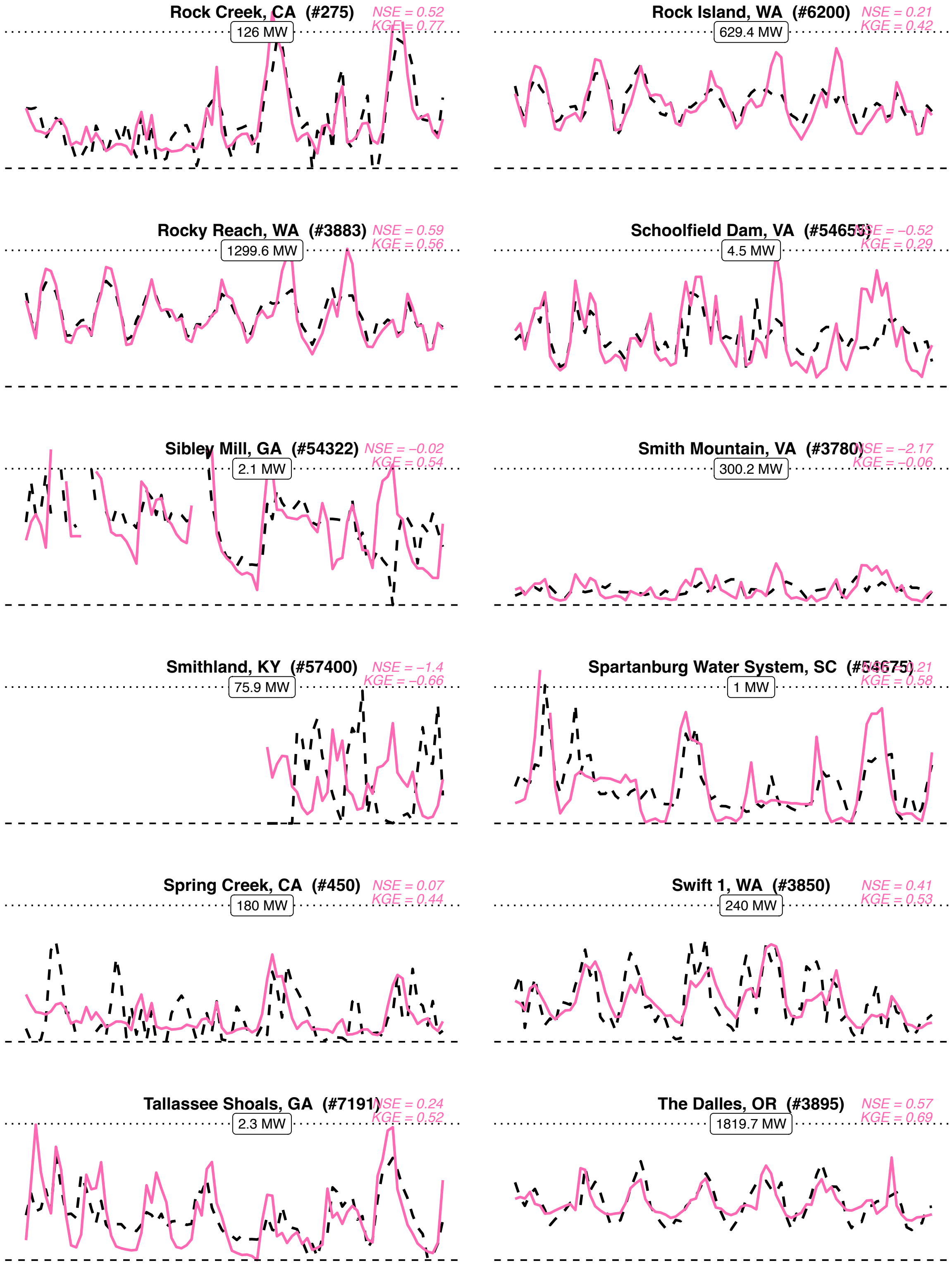

Model validation (downstream flow based) continued.

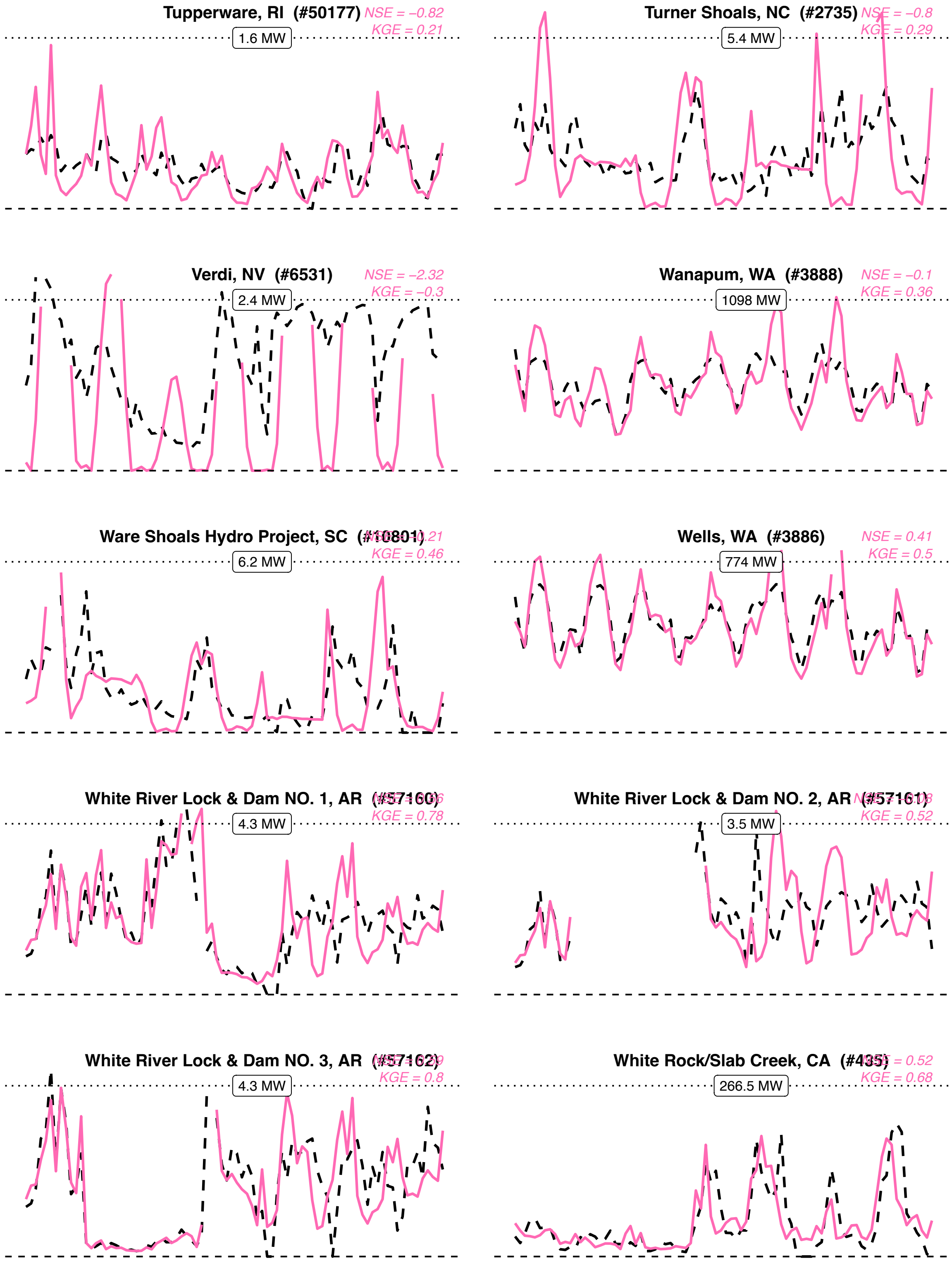

Supplement: Supplementary file 1 — Supplementary Information [file 41597_2022_1748_MOESM1_ESM.pdf]
